# Effect of 6-week karate (kihon) and basic movement exercise on balance performance in visually impaired individuals

**DOI:** 10.3389/fphys.2023.1332393

**Published:** 2023-12-14

**Authors:** Aylin Özge Pekel, Ceren Suveren, Yasin Arslan, Belma Yavaşoğlu, Ayşegül Beykumül, Tebessüm Ayyıldız Durhan, Levent Ceylan

**Affiliations:** ^1^ Faculty of Sports Sciences, Gazi University, Ankara, Türkiye; ^2^ Health Sciences Institute, Kırşehir Ahi Evran University, Kırşehir, Türkiye; ^3^ Turgut Özal Medical Center, İnönü University, Malatya, Türkiye; ^4^ Faculty of Sports Sciences, Sivas Cumhuriyet University, Sivas, Türkiye

**Keywords:** karate, basic movement exercise, balance, visually impaired, performance

## Abstract

Today, the participation of visually impaired individuals in sports activities is essential. Because the ability to move independently starts to develop with a delay in visually impaired individuals, physical activity is necessary to compensate for developmental delay, eliminate the problem of independent movement in social life by reducing obstacle perception problems, develop self-confidence, and provide regular muscle activation and motivation to move. The study investigated the effect of 6 weeks of karate (kihon) and basic movement training on balance performance in individuals with congenital visual impairment. Fifteen visually impaired individuals aged between 10 and 14 participated in the study, and three groups were formed: experiment 1, experiment 2, and the control group. After the pre-tests were taken, the experimental groups received karate and basic movement training in addition to physical education classes for 6 weeks, while the control group received only physical education classes. When the study results were examined, there were highly significant differences between the pre- and post-test values of the groups receiving karate and basic movement training. At the same time, no progress was observed in the control group. In the post-test comparison of the karate and control groups, positive significance was found in the values of the karate group. In the same way, in the post-test comparison of the basic movement training group and the control group, positive progress was made in the basic movement training group. The post-test comparison of the basic movement training and karate groups was the same. As a result, it was concluded that basic movement training and karate exercises applied for 6 weeks positively affected the balance development in visually impaired individuals aged 10–14 years. No difference was found between the exercise protocols regarding effectiveness, and no improvement was observed in individuals who did not participate in any exercise.

## 1 Introduction

In daily life, 80% of the information we provide comes from visual stimuli (Cengizel et al., 2022). Visual, vestibular, and proprioceptive systems must work together for movement perception. Concepts such as depth, distance, and proximity directly affect the maintenance of balance or restoration in case of loss (Kalia et al., 2010; Winnick et al., 2017; Altunay et al., 2022). Meinel and Schnabel define balance skill as the capacity to maintain balance and manage one’s position during and after changes in body position (Erkmen et al., 2007). Balance control is a complex motor skill that involves the integration of sensory information and the planning and execution of adaptive movement patterns. Balance can be divided into two types: static and dynamic. Static balance is concerned with maintaining the body’s center of gravity in situations of minimal movement. In contrast, dynamic balance focuses on achieving or maintaining balance during specific activities or on unstable surfaces (Hrysomallis, 2011).

Balance is a crucial component in various athletic skills, such as changing direction, stopping, starting, holding position, manipulating objects, and maintaining specific body postures, and is widely recognized to play a vital role in these skills. Balance, which plays an influential role in overcoming or minimizing movement limitations, is a skill that directly affects the body’s ability to move. Without the sense of sight, balance development lags due to lack of experience (Casselbrant et al., 2007). Therefore, sports training is crucial for visually impaired individuals (Suveren-Erdogan and Suveren, 2018). Lack of balance can lead to difficulties in visually impaired individuals, such as various walking styles, such as extending their hands forward or taking cautious, slow steps to avoid accidents (Arslantekin, 2014).

Balance quality is directly related to the information the vestibular system provides and optic flow (Casselbrant et al., 2007). The absence or reduced functionality of the sense of vision causes other sensory organs to become sensitized to compensate for this deficiency. However, the lack of the importance of sight can never be fully compensated for by other senses (Cengizel et al., 2022). Individuals with disabilities need sufficient spatial and physical awareness as well as socialization and motor skills such as endurance, strength, balance, and coordination to move independently and competently (Kalia et al., 2010; Winnick et al., 2017; Altunay et al., 2022). Participating in physical activity is crucial to compensate for developmental delays, improve the perception of disability, increase self-confidence, and promote regular muscle activation (Sarimski, K. (1997). It also serves as a motivating factor for greater mobility in social life (Christiansen et al., 2010).

Special physical programs should be designed to meet the unique needs of visually impaired individuals. These adapted programs should strive to provide a corrective, competent, safe, personally satisfying, and successful experience (Winnick et al., 2017). Such initiatives support the optimal personal development and self-actualization of individuals with visual impairment, ultimately improving their quality of life (Murray and Gallahue, 1987). Basic movement training, which is very important for individuals’ motor development and motor performance, also refers to the perfect execution of basic movements at the most superficial level. This means that the balance skill is at an adequate level. The most crucial step in the transition from the motor development period to the motor performance process is the proper application of fundamental movement skills. Kihon training means the basic training of karate. It is a training system that develops the ability to be aware of the whole and parts of the body and to reflect this awareness to the movement skills at a high level, and at the same time, allows the application of versatile movement practices with small displacements.

Considering the mobility skills of visually impaired individuals and the need to design special physical programs to meet their needs (Winnick et al., 2017), basic movement training and kihon training are very functional in terms of balance development in visually impaired individuals. However, although many studies show that karate positively affects balance performance, the scarcity of studies on visually impaired individuals has attracted attention. For this reason, it was preferred for visually impaired individuals to receive basic movement and kihon training for their balance development. It aimed to examine the effect of balance levels of individuals with visual impairment at B2 and B3 levels as a result of 6 weeks of training. The contribution of basic movement training and karate sport, a new field in which visually impaired individuals can participate, to the balance status of individuals with special needs reveals the unique value of our research.

## 2 Material and methods

### 2.1 Participants

Fifteen visually impaired individuals between the ages of 10 and 14, all in their early adolescence, participated in the study. Participants were randomly divided into three groups: experiment 1 (five subjects, primary movement group; three girls and two boys), experiment 2 (five subjects, karate group; two girls and three boys), and control (five subjects: three girls and two boys). The sample group was selected by using a readily available sampling method, and no power analysis or gender discrimination was performed due to the small number of visually impaired subjects. Goalball Googles are sports equipment used in blind and visually impaired participation sports and other team games where full light dimming is required to restrict the vision of all athletes and ensure fair play for all. These goggles provide both protection and full light dimming thanks to the raised foam inserts around the eye socket, while at the same time protecting important parts of the face such as the forehead and cheekbones with a wide nosepiece. These goggles have an aerodynamic strap system for a better fit on the head (https://goalfixsports.com). During the study, the subjects continued to work with Goalball Googles throughout the entire training process and during all measurements so that light perception would not affect the learning processes of the subjects differently.

The study included individuals with visual impairment levels in the B2 and B3 categories, aged between 10 and 14 years, with no health problems other than visual impairment, and who had not participated in sports activities other than physical education. Children who had a bodily injury or surgery in the last 6 months, who did not participate in 10% of the total number of training sessions and/or any of the measurements, and who voluntarily withdrew from the study at any time during its course were excluded.

The 15 visually impaired individuals who participated in the study were selected from categories B1, B2, and B3 according to the U.S. Association of Blind Athletes (USABA) classification system, which is explained in Table 1.

**TABLE 1:** Classification of the USABA (U.S. Association of Blind Athletes).

**B1:** Even if there is light perception in both eyes, there is no vision. The subject cannot recognize the shape of a hand from any distance or direction
**B2:** The angle of vision is 5 degrees or less than 5 degrees. Visual acuity is no better than 20/600. In other words, the subject can see the shape of the hand from a distance of approximately 30 cm
**B3:** Angles of vision are between 5 and 20º. Visual acuity is 20/600–60/600. In other words, the subject can see the shape of the hand from a distance of approximately 30 cm to 1 m

### 2.2 Experimental design

Experimental groups 1 (basic movement training) and 2 (karate kihon) were subjected to daily karate and basic movement training for 1 h, 2 days a week for 6 weeks. The control group participated only in physical education classes at school.

Before the training, the athletes were informed about karate and basic movement training and were told how to perform the movements correctly and what to pay attention to. The training exercises took place simultaneously every day after a 10-min warm-up. During the training, the instructors provided physical and verbal stimulation to the students while having them perform the movements. They had the experimental group practice all the activities in the programs. The basic movement training was conducted by senior gymnastics coach, Ceren Suveren, one of the researchers in the study, and the karate kihon training was conducted by Senior karate coach, Belma Yavaşoğlu (3rd Dan), one of the researchers in the study. Training protocols and measurements were carried out in the gymnasium of the school for the visually impaired, which was specially prepared for the disabled, with columns and walls covered with soft cushions, and with a rubber floor, accompanied by experts in the field.

#### 2.2.1 Basic movement training program

**Table:** 

• Basic movement exercise protocol		• Repetition	• Set
• 1	• Squat	• 15	• 2
• 2	• Open leg forward–backward stretch exercise	• 10	• 2
• 3	• Stand on a single foot	• 10 × 2 (Left–right)	• 2
• 4	• Left–right pendulum movement	• 10 × 2 (Left–right)	• 2
• 5	• Left–right leg plane position	• 10 × 2 (Left–right)	• 2

*For motion application, see https://doi.org/10.5539/jel.v7n6p191.

#### 2.2.2 Karate–Do kihon training program

**Table:** 

• Karate–Do kihon exercise protocol		• Repetition	• Rest
• 1st week	• Yoi and Seiken Tsuki	• 10 × 3	• 1 min
• 2nd week	• Oi Tsuki Chudan	10 × 3	1 min
• 3rd week	• Oi Tsuki Codan	10 × 3	1 min
• 4th week	• Age Uke	10 × 3	1 min
• 5th week	• Uchi Uke	10 × 3	1 min
• 6th week	• Mae Geri	10 × 3	1 min

*For training application, see https://doi.org/10.33438/ijdshs.1293669.

### 2.3 Test protocol

#### 2.3.1 Y-balance test

The material for the dynamic balance test consists of three PVC pipes with anterior, posteromedial, and posterolateral expansion directions connected to a central platform (balance point) and three PVC blocks inserted into these pipes. The tubes on the posterior sides of the platform (posteromedial and posterolateral) are spaced 45° from each other and 135° from the tube in the anterior direction. To determine the distance that can be reached by the athletes, distance gages are attached to the tubes connected to the platform in the anterior, posteromedial, and posterolateral directions at 1-mm intervals (Türkeri et al., 2020).

Application protocol of the dynamic Y-balance test of the lower extremities: The lower extremity dynamic Y-balance test was performed on the same day and time with an interval of 2 weeks. The test was performed separately on both feet of the subjects (dominant and non-dominant). The subjects, wearing sports clothes that did not restrict their movements, stood with bare feet on the platform of the dynamic Y-balance test, holding their hands near the waist and their feet in the middle. Then, while maintaining a firm stance with one foot, the subject pushed the blocks with the toe in the anterior (0°), posteromedial (45°), and posterolateral (45°) directions with the other foot (bringing them back to the fixed stance without touching the ground each time). After repeating the test three times in all three directions (anterior, posteromedial, and posterolateral), the mean values of the measurements were determined, and the normalization formula was used (Lai et al., 2017; Türkeri et al., 2020).

Application protocol for the dynamic Y-balance test of the upper extremities: The test was performed on both arms of the subjects (dominant and non-dominant). The subjects balance in a push-up position (anterior position) on the Y-balance test platform with their hands fixed on the center point and wearing sportswear that does not restrict their movements. At the same time, their feet are shoulder-width apart, and their legs and hip center are fixed together. The athlete then holds a firm stance with one hand, grips with the upper extremity only, without support from the lower extremity and hip center, and points the finger in the medial (0°), inferolateral (45°) (from inside under the other hand), and superolateral (45°) directions. With the tip of the finger, the subject nudges the blocks. Each time, the athlete performed the exercise by returning the hand to a firm stance without touching the floor. After the test was repeated twice in all three directions (medial, inferolateral, and superolateral), the mean values of the measurements were taken, and the normalization formula was used (Gorman et al., 2012).

The visually impaired subjects were assisted at the beginning of the test during the measurements so that they could maintain control and the test could be performed without errors (Türkeri et al., 2020).

#### 2.3.2 Heel–toe step test

After being verbally informed of the study to be conducted, visually impaired participants were asked to walk from heel to toe on a 10-meter line, with the heel of the foot touching the toe of the foot on the ground at each step, accompanied by an audio stimulus to detect spatial orientations and loss of balance. Total walking error values were recorded at the end of the test (Bruininks, 1978; Suveren-Erdogan and Suveren, 2018).

#### 2.3.3 Flamingo balance test

The static balance measurements of the subjects participating in the study were measured using a flamingo balance beam. The equipment has a beam 50 cm long, 4 cm high, and 3 cm wide The subjects stood on the wide wooden balance beam and attempted to maintain balance for 1 min. Time recording was stopped when the balance was disturbed (when the subject let go of his/her foot while holding on, fell off the board, touched the floor with any part of his/her body, etc.). When the subjects regained their balance by climbing the balance beam, the time was continued where it was left off. The test continued in this manner for 1 minute. At the end of the time (1 min), each attempt by the test group to maintain balance (after a fall) was counted, and this number (number of falls) was recorded as the test group’s score at the end of the 1-minute test period. The test was performed with the dominant and non-dominant foot (Barabas et al., 1996; Suveren et al., 2017; Groselj et al., 2019; Sember et al., 2020).

### 2.4 Statistical methodology

All subjects participating in the study were administered pre- and post-tests for all test protocols before and after the exercises. SigmaPlot 11.0 software was used to analyze the data. To compare pre- and post-tests, a paired t-test was used for parametric distributed data and a Wilcoxon test was used for non-parametric distributed data. When comparing three independent groups, the one-way repeated measures test was used for parametric distributed data and the Friedman test was used for non-parametric distributed data (Oz et al., 2010; Yavasoglu and Suveren-Erdogan, 2023).

## 3 Result

As seen in Table 2, when the within-group pre-test values of static and dynamic balance parameters of karate, basic movement, and control groups are compared, it can be seen that the groups are homogeneously distributed. When the within-group post-test values were compared, a statistically significant difference was found for all parameters, except for the UE-IL-R correct dynamic balance value of the karate group and the LE, PL-L, UE-MD-L, and IL-L dynamic balance parameters of the basic movement group. No significant difference was found between the values before and after the test of the control group.

**TABLE 2:** Analysis of static and dynamic balance parameters’ pre-test and post-test values in karate, basic movement, and control groups.

	Karate group				Basic movement group				Control group			
	(*n* = 5)				(*n* = 5)				(*n* = 5)			
Balance parameters	Pre-test	Post-test			Pre-test	Post-test			Pre-test	Post-test		
	x ± sd	x ± sd	p	% Difference	x ± sd	x ± sd	p	% Difference	x ± sd	x ± sd	p	% Difference
SB	25,60 ± 3,05	17,8 **±** 1,92	<0,001	−.30,4	25 **±** 2,73	18,8 **±** 2,38	<0,001	−.24,8	25,0 **±** 3,39	24,8 **±** 3,34	0,62	−.0,8
DB—LE												
ANT-R	74,0 **±** 2,91	81,4 **±** 4,39	0,001	.10	74 **±** 2,73	80,8 **±** 3,70	<0,001	.9,18	74,2 **±** 3,34	73,6 **±** 3,57	0,25	−.0,8
ANT-L	81,4 **±** 2,30	87,6 **±** 2,19	<0,001	.7,6	81,2 **±** 2,58	87,4 **±** 3,36	<0,001	.7,6	83,2 **±** 2,38	83,6 **±** 2,07	0,50	.0.4
PL—R	68,6 **±** 2,96	77,0 **±** 2,91	0,005	.12,2	67,2 **±** 4,32	73,8 **±** 5,21	<0,001	.9,8	65,6 **±** 4,93	66,6 **±** 4,61	0,08	.1,5
PL—L	94,6 **±** 2,96	102,2 **±** 3,56	0,001	.8	92,6 **±** 3,50	100,8 **±** 4,55	0,063	.8,8	93 **±** 3,87	93,2 **±** 3,63	1,00	.1
PM—R	103 **±** 2,12	112,4 **±** 3,36	0,004	.9,1	103,2 ± 2,58	111 **±** 2,73	<0,001	.7,5	103,2 **±** 2,58	103,2 **±** 2,58	1,00	.0
PM—L	92,4 **±** 2,30	103,2 **±** 1,92	<0,001	.11,6	92,4 **±** 2,88	98,6 **±** 3,91	<0,001	.6,7	92,6 **±** 2,40	92,8 **±** 1,78	0,62	.0,2
DB—UE												
MD—R	104 **±** 3,80	114,0 **±** 4,18	<0,001	.9,6	102,8 **±** 3,56	110 **±** 5,0	<0,001	.7	103,0 **±** 2,73	103,4 **±** 2,96	0,50	.0,3
MD—L	57,0 **±** 1,58	65,8 **±** 2,77	<0,001	.15,4	57,2 **±** 1,92	65,4 **±** 1,51	0,063	.14,3	58 **±** 3,16	58,4 **±** 3,64	0,50	.1,72
IL—R	54,6 **±** 2,30	63,6 **±** 3,05	0,063	.16,4	53,4 **±** 2,51	59,6 **±** 4,39	0,008	.11,6	53,4 **±** 2,07	53,4 **±** 2,07	1,00	.0
IL—L	83,0 **±** 2,55	90,4 **±** 3,05	<0,001	.8,9	83,4 **±** 2,40	90,8 **±** 3,11	0,063	.8,8	82,6 **±** 2,40	83 **±** 2	0,50	.0,4
SL—R	64,8 **±** 2,86	72,4 **±** 3,97	<0,001	.11,7	64 **±** 2,73	71,2 **±** 3,56	<0,001	.11.2	64,6 **±** 3,20	64,8 **±** 2,86	1,00	.0,3
SL—L	66,6 **±** 2,40	75,8 **±** 2,28	0,002	.13,8	65,4 **±** 3,20	72,6 **±** 3,05	<0,001	.11	65,4 **±** 4,03	66,2 **±** 3,76	0,12	.1,22
HT	5,2 **±** 1,92	3,2 **±** 1,30	0,011	−.38	5,4 **±** 2,30	3,2 **±** 1,09	0,020	−.40,7	6 **±** 2,23	5,4 **±** 1,81	0,25	−.10

*p* < 0,05.

SB, static balance (number of falls); DB, dynamic balance; LE, lower extremity; UE, upper extremity; ANT-R, anterior right; ANT-L, anterior left; PL-R, posterolateral right; PL-L, posterolateral left; PM-R, posteromedial right; PM-L, posteromedial left; MD-R, medial right; MD-L, medial left; IL-R, inferolateral right; IL-L, inferolateral left; SL-R, superolateral right; SL-L, superolateral left; HT, heel–toe (error score).

As seen in Table 3, the pre-test and post-test values of static and dynamic balance parameters of karate, basic movement training, and control groups were compared, and no significant difference was found between the pre-test values. However, when the post-test values were examined, a significant positive difference was found in all parameters, except for a single parameter (DB-ANT-L).

**TABLE 3:** Analysis of static and dynamic balance parameters’ pre-test and post-test values in the karate, basic movement, and control groups.

	Pre-test				Post-test			
	(*n* = 5)				(*n* = 5)			
Balance parameters	Karate group	Basic movement group	Control group		Karate group	Basic movement group	Control group	
	x ± sd	x ± sd	x ± sd	p	x ± sd	x ± sd	x ± sd	p
SB	25,6 ± 3,05	25,0 **±** 2,73	25,0 **±** 3,39	0,600	17,8 ± 1,92	18,8 **±** 2,38	24,8 **±** 3,34	0,002
DB-LE								
ANT-R	74,0 ± 2,91	74,0 **±** 2,73	74,2 **±** 3,34	0,991	81,4 ± 4,39	80,8 **±** 3,70	73,6 **±** 3,57	0,014
ANT-L	81,4 ± 2,30	81,2 **±** 2,58	83,2 **±** 2,38	0,482	87,6 ± 2,19	87,4 **±** 3,36	83,6 **±** 2,07	0,055
PL-R	68,6 ± 2,96	67,2 **±** 4,32	65,6 **±** 4,93	0,616	77,0 ± 2,91	73,8 **±** 5,21	66,6 **±** 4,61	0,008
PL—L	9,6 ± 2,96	92,6 **±** 3,50	93,0 **±** 3,87	0,695	102,2 ± 3,56	100,8 **±** 4,55	93,2 **±** 3,63	0,008
PM-R	103,0 ± 2,12	103,2 **±** 2,58	103,2 **±** 2,58	0,992	112,4 ± 3,36	111,0 **±** 2,73	103,2 **±** 2,58	<0,001
PM-L	92, 4 ± 2,30	92,4 **±** 2,88	92,6 **±** 2,40	0,983	103,2 ± 1,92	98,6 **±** 3,91	92,8 **±** 1,78	<0,001
DB-UE								
MD-R	104,0 ± 3,80	102,8 **±** 3,56	103,0 **±** 2,73	0,820	114,0 ± 4,18	110,0 **±** 5,00	103,4 **±** 2,96	0,005
MD-L	57,0 ± 1,58	57,2 **±** 1,92	58.0 **±** 3,16	0,835	65,8 ± 2,77	65,4 **±** 1,51	58,4 **±** 3,64	0,002
IL-R	54,6 ± 2,30	53,4 **±** 2,51	53,4 **±** 2,07	0,660	63,6 ± 3,05	59,6 **±** 4,39	53,4 **±** 2,07	0,001
IL-L	83,0 ± 2,55	83,4 **±** 2,40	82,6 **±** 2,40	0,906	90,4 ± 3,05	90,8 **±** 3,11	83,0 **±** 2,00	0,001
SL-R	64,8 ± 2,86	64,0 **±** 2,73	64,6 **±** 3,20	0,954	72,4 ± 3,97	71,2 **±** 3,56	64,8 **±** 2,86	0,010
SL-L	66,6 ± 2,40	65,4 **±** 3,20	65,4 **±** 4,03	0,855	75,8 ± 2,28	72,6 **±** 3,05	66,2 **±** 3,76	0,001
HT	5,20 ± 1,92	5,4 **±** 2.30	6,00 **±** 2,23	0,865	3,20 ± 1,30	3,20 **±** 1,09	5,40 **±** 1,81	0,049

*p* < 0,05.

SB, static balance (number of falls); DB, dynamic balance; LE, lower extremity; UE, upper extremity; ANT-R, anterior right; ANT-L, anterior left; PL-R, posterolateral right; PL-L, posterolateral left; PM-R, posteromedial right; PM-L, posteromedial left; MD-R, medial right; MD-L, medial left; IL-R, inferolateral right; IL-L, inferolateral left; SL-R, superolateral right; SL-L, superolateral left; HT, heel–toe (error score).

## 4 Discussion

The movement skills of visually impaired individuals may develop differently from those of their peers. One of the most important reasons for this is problems in the perception of disability and the protective attitude of the families. The different perception of the outside world also causes visually impaired individuals to perform movements in different body shapes than average. For example, they may walk with their head forward, back bent, and knee joints fixed (Suveren-Erdoğan et al., 2018; Suveren-Erdoğan et al., 2018; Bennett et al., 2019; Cengizel et al., 2022). This situation may lead to difficulty or even loss of balance when shifting the center of gravity. Loss of balance, especially when walking, negatively affects the lives of the visually impaired and leads to disruptions in their social lives (Montarzino et al., 2007; Ray et al., 2008; Coughlan et al., 2012). More accurate body positioning is one of the critical factors affecting balance. Therefore, balance occupies an essential place in the lives of visually impaired individuals. The development of balance skills in visually impaired individuals, which are similar to those of individuals with sight, depends on the training content (Anthony et al., 2002).

The reason for choosing karate as a branch besides essential movement exercise in the study’s design is the coordination requirement resulting from the simultaneous movement of arms, trunks, and legs in different positions while performing the exercises. In this way, the balance development in disabled individuals should be ensured during the learning phase of the exercises when practicing skills that require different body usage. At this point, the significance level in favor of the experimental groups in the post-test comparison between the experimental and control groups shows that the study achieved its objective. While positive significance was found for both experimental groups compared to the control groups, the fact that no significant difference was found when comparing the pre-test and post-test values of the karate group and the basic movement group can be interpreted to mean that both protocols positively affect the balance performance in visually impaired individuals. The fact that the balance values of the control group did not change even though they participated in physical education suggests that disabled individuals need specially planned exercises for balance development.

The literature confirms that exercises reduce the adverse effects of falls and injuries due to loss of balance (Rubenstein et al., 1994; Lord and Dayhew, 2001; Lee and Scudds, 2003; Ray et al., 2008; Jazi et al., 2012). For this reason, the effect of different exercise protocols on balance development is of great importance, both in contributing to the motor development processes in visually impaired individuals and in observing balance development more specifically. A literature review reveals that several applications for visually impaired individuals have been developed. Cheung et al. (2008) concluded that the experimental group showed significant improvement in balance development compared to the control group due to the 12-week exercise protocol they used with the elderly (Cheung et al., 2008). Aki et al. (2007) achieved progress in many areas, including balance, by applying a motor training program to train visually impaired children (Aki et al., 2007).

Two studies examined the effects of some basic standing postures and wall-based basic movement training on visually impaired individuals’ walking skills and balance levels. Examination of the study results revealed an improvement in the walking quality in visually impaired individuals, an increase in the ability to maintain balance at walking speed, and a decrease in loss of balance. When the measurement methods, exercise protocols, and results of the studies are evaluated, they are found to be similar to the results of previous research (Suveren-Erdogan et al., 2018; Suveren-Erdogan and Suveren, 2018). Another study highlighted the importance of teaching basic postures, body awareness, and spatial orientation in teaching movement and sports skills to visually impaired individuals (Suveren-Erdogan et al., 2018).

In this study, no significant difference was found between the pre-test values when comparing the pre-test and post-test values of the static and dynamic balance parameters of the primary movement group and the control group. Still, when examining the post-test values, a significant positive difference was observed in all parameters, except for a single parameter (Y-balance lower anterior left). The results of the study are consistent with those reported in the literature. In another study that researched balance development in visually impaired individuals, 8-week balance training was conducted in visually impaired children to monitor the development of dynamic balance, and the experimental group showed significant improvement in both within-group and between-group comparisons of balance performance (Jazi et al., 2012). Akyol et al. (2017) examined the effects of exercise on balance and walking distance in a study on athletes and sedentary visually impaired individuals (Akyol et al., 2017). The researchers found that the walking distance and balance status of national athletes were more significant at the *p* < 0.001 level than those of sedentary visually impaired individuals, and consistent with this finding, they suggested that visually impaired individuals may be more independent in their daily activities if they incorporate sports into their lives.

In a study conducted with goalball and track and field athletes, similar to this study, the technique of brisk walking was used to test dynamic balance performance (Seok-Min et al., 2010). Kıral (2007) found a statistically significant improvement in the experimental group compared to the control group as a result of balance tests in a study conducted with visually impaired individuals who do and do not participate in sports (* *p* 0.05*) (Kıral, 2007). Chen et al. (2012) concluded that Tai Chi exercises, which require balance and coordination similar to the kata branch in karate, have a positive effect on balance development (*p* = 0.024) (Chen et al., 2012). Their results support the effectiveness of the karate techniques used in the study. Suveren and Yavaşoğlu (2023) investigated the effects of short-term karate training on some parameters of physical fitness in visually impaired individuals. They concluded that kihon training of visually impaired individuals improves the physical fitness in visually impaired individuals (Yavasoglu and Suveren-Erdoğan, 2023).

In this study, when the static and dynamic balance parameters of the karate and the control groups were compared in terms of pre-test and post-test values, there was no significant difference between the pre-test values. However, when the post-test values were examined, there was a significant positive difference in all parameters except for a single parameter (10 m heel to toe test). The study is similar to the literature regarding the results obtained and the exercise protocol.

As a result, it was found that basic movement training and karate exercises performed for 6 weeks positively affected balance development in visually impaired subjects aged 10–14 years. There was no difference in efficacy between exercise protocols, and no improvement was seen in individuals who did not participate in any exercise. The literature review indicates that exercise positively affects the development of balance and walking skills in visually impaired individuals. This study contributes to the literature on this topic.

### 4.1 Limitation

Under present conditions, it is not easy to reach the visually impaired population. It is tough to find subjects in the same age group and with similar levels of disability, especially when conducting long-term exercise protocols. For this reason, the number of experimental and control groups could be fixed at 5.

Compared to individuals without disabilities, visually impaired individuals have less training continuity due to external factors such as illness, pain, disability, or accidents. Considering the optimum time interval in which the groups in the study could continue training, the study was limited to 6 weeks.

## Data Availability

The original contributions presented in the study are included in the article/Supplementary Material; further inquiries can be directed to the corresponding author.
